# Transient Expression of Reck Under Hepatic Ischemia/Reperfusion Conditions Is Associated with Mapk Signaling Pathways

**DOI:** 10.3390/biom10050747

**Published:** 2020-05-11

**Authors:** Andrea Ferrigno, Laura G. Di Pasqua, Giuseppina Palladini, Clarissa Berardo, Roberta Verta, Plinio Richelmi, Stefano Perlini, Debora Collotta, Massimo Collino, Mariapia Vairetti

**Affiliations:** 1 Department of Internal Medicine and Therapeutics, University of Pavia, 27100 Pavia, Italy; andrea.ferrigno@unipv.it (A.F.); lauragiuseppin.dipasqua01@universitadipavia.it (L.G.D.P.); giuseppina.palladini@unipv.it (G.P.); clarissa.berardo01@universitadipavia.it (C.B.); plinio.richelmi@unipv.it (P.R.); stefano.perlini@unipv.it (S.P.); 2 Fondazione IRCCS Policlinico San Matteo, 27100 Pavia, Italy; 3 Department of Drug Science and Technology, University of Turin, 10125 Turin, Italy; roberta.verta@unito.it (R.V.); debora.collotta@unito.it (D.C.); massimo.collino@unito.it (M.C.); 4 Emergency Department, Fondazione IRCCS Policlinico San Matteo, 27100 Pavia, Italy

**Keywords:** RECK, matrix metalloproteinase, MAPKs, ischemia/reperfusion, eNOS, iNOS

## Abstract

In this study, we demonstrated the involvement of matrix metalloproteinases (MMPs) in hepatic ischemia/reperfusion (I/R) injury. Our aim is to evaluate the impact of reperfusion on I/R-related changes in RECK, an MMP modulator, and mitogen-activated protein kinase (MAPKs) pathways (ERK, p38, and JNK). Male Wistar rats were either subjected to 60 min partial-hepatic ischemia or sham-operated. After a 60 min or 120 min reperfusion, liver samples were collected for analysis of MMP-2 and MMP-9 by zymography and RECK, TIMP-1, and TIMP-2 content, MAPKs activation (ERK1/2, JNK1/2, and p38), as well as iNOS and eNOS by Western blot. Serum enzymes AST, ALT, and alkaline-phosphatase were quantified. A transitory decrease in hepatic RECK and TIMPs was associated with a transitory increase in both MMP-2 and MMP-9 activity and a robust activation of ERK1/2, JNK1/2, and p38 were detected at 60 min reperfusion. Hepatic expression of iNOS was maximally upregulated at 120 min reperfusion. An increase in eNOS was detected at 120 min reperfusion. I/R evoked significant hepatic injury in a time-dependent manner. These findings provide new insights into the underlying molecular mechanisms of reperfusion in inducing hepatic injury: a transitory decrease in RECK and TIMPs and increases in both MAPK and MMP activity suggest their role as triggering factors of the organ dysfunction.

## 1. Introduction

Hepatic ischemia/reperfusion (I/R) injury may occur in a variety of clinical situations, including transplantation, liver resection, trauma, and vascular surgery. The reperfusion after ischemia may trigger I/R injury exacerbating cellular damage. The effects of reperfusion include excessive production of reactive oxygen species, oxidative stress, mitochondrial dysfunction, and systemic inflammatory response. Neutrophils play a critical role during the initial phase of I/R (0–6 h) triggering activation of pro-inflammatory intracellular cascades. One of the best characterized pathways within the context of organ I/R injury is the mitogen-activated protein kinase (MAPKs) family [1]. Among MAPKs, p38, JNK1/2, and ERK 1/2, activated by a variety of cellular stressors including I/R, could potentially serve as potential targets in hepatic I/R injury [2,3].

Hepatic I/R injury is also connected with matrix metalloproteinases (MMPs) gene expression, activation, and release concomitant with alterations of tissue integrity [4]. The MMPs are a family of 24 proteases using zinc-dependent catalysis to breakdown extracellular matrix (ECM) components, allowing cell movement and tissue reorganization. Furthermore, MMP-2 and MMP-9 play a crucial role as they are the main components of Disse’s space composed of loose extracellular matrix (ECM) [5]. We previously demonstrated that dysregulated expression and activation of MMPs are involved in hepatic injury and that changes in MMP activities already occur during the early phases of reperfusion [6]. Among different MMPs, MMP-9 is emerging as an important mediator of leukocyte traffic in hepatic I/R [7]. MMP-9 plays a crucial role in leukocyte recruitment and activation leading to liver I/R damage [8]. MMP-9(-/-) mice and mice treated with an anti-MMP-9 antibody showed significantly reduced I/R damage, altered neutrophil migration, and depressed myeloperoxidase (MPO) activation [8]. Of note, the regulation of MMP activity appears as a multifaceted process; in particular, the relationship between the production of nitric oxide (NO) by inducible NO synthase (iNOS) and MMP-9 induction has obtained much attention over the last few years [9]. Selective iNOS inhibition down-regulates MMP-9 activity and disrupts leukocyte migration in hepatic I/R injury [10]. Most recent studies demonstrate that the activity of MMP-2 or MMP-9 is reduced by interaction with the reversion-inducing-cysteine-rich protein with kazal motifs (RECK) [11,12]. Recently, a study discovered RECK isoforms with opposing effects on cell migration: the short isoform appears to compete with MMP-9 for binding to the long RECK isoform [13]. RECK was identified as a transformation-suppressor gene which, regulating the expression of several MMPs, is involved in the inhibition of the tumor invasion and metastasis process [14]. RECK contains a glycosylphosphatidylinositol domain and a number of cysteine residues. It presents differences in the amino acid sequence when compared with the tissue inhibitors of metalloproteinases (TIMPs), which makes RECK an innovative membrane-targeted MMP regulator [14]. Besides, our understanding of the time course of activation of signaling cascades exerting a key role in liver injury following reperfusion remains incomplete and warrants further investigation. 

Thus, the aim of the present study is to evaluate the impact of reperfusion on RECK and MAPK signaling pathways under hepatic I/R conditions. Their correlations with markers of organ dysfunction, such as MMP activity, iNOS, and eNOS were further investigated to better describe pathogenic mechanism(s) suitable for pharmacological modulation. 

## 2. Materials and Methods

### 2.1. Materials 

All reagents were of the highest grade of purity available and were obtained from Sigma-Aldrich (Milan, Italy). 

### 2.2. Animals and Experimental Design

Male Wistar rats (Harlan-Nossan, Italy) were used in this study. The animals were allowed free access to water and food in all the experiments. The use and care of animals in this experimental study was approved by the Italian Ministry of Health and by the University of Pavia Commission for Animal Care (Document number 179/2017-PR). The effects of I/R were studied in vivo in a partial normothermic hepatic I/R model (n = 18). The rats were anesthetized with sodium pentobarbital (40 mg/kg i.p.), the abdomen was opened via a midline incision. Ischemia to the left and the median lobe was induced for 60 min with microvascular clips by clamping the branch of portal vein and the branch of the hepatic artery after the bifurcation to the right lobe, with the abdomen temporarily closed with a suture [6]. After 60 min of ischemia, the abdomen was reopened, the clips were removed, the abdomen was closed again, and the liver was allowed to reperfuse for 60 or 120 min. By using partial, rather than total, hepatic ischemia, portal vein congestion and subsequent bacterial translocation into the portal venous blood was avoided. Sham animals were subjected to the same procedure without clamping the vessels (n = 12). To prevent postsurgical dehydration and hypotension, 1 mL of saline was injected into the inferior vena cava. All the animals were maintained on a warm support to prevent heat loss with rectal temperature at 37 ± 0.1°C. Animals were sacrificed under general anesthesia by exsanguination. Blood was drawn from the vena cava and allowed to clot at room temperature. After 15 min serum was centrifuged at 3000× *g* for 10 min at 4°C. At the end of ischemia or after 60 min or 120 min reperfusion, hepatic biopsies were quickly removed from the median lobe and immediately frozen in liquid nitrogen, as were serum samples. 

### 2.3. Biochemical Assays 

Liver injury was assessed by serum levels of alanine transaminase (ALT), aspartate transaminase (AST), and alkaline phosphatase (ALP). Total and direct bilirubin were quantified using standard commercial kits (Merck, Italy). 

### 2.4. Gelatin Zymography 

Protein extraction from snap-frozen samples and gelatin zymography were performed as described previously [15]. To detect MMP lytic activity samples were homogenized in an ice-cold extraction buffer and protein content was normalized by a final concentration of 400 μg/mL in the sample loading buffer (0.25 M Tris-HCl, 4% sucrose *w*/*v*, 10% SDS *w*/*v*, and 0.1% bromphenol blue *w*/*v*, pH 6.8). After dilution, the samples were loaded onto electrophoretic gels (SDS-PAGE), containing gelatin, under nonreducing conditions. Following the electrophoretic run, the gels were washed twice and were incubated at 37 °C. To reveal zones of lysis, gels were stained with Coomassie Blue. The zymograms were analyzed by a densitometer (GS 900 Densitometer BIORAD, Hercules, CA, USA), and proteinases activity was expressed as optical density (OD), reported to 1 mg/mL protein content.

### 2.5. Western Blot Assay 

Liver tissue samples were homogenized in an ice-cold Lysis Buffer supplemented with Protease Inhibitor Cocktail and centrifuged at 15,000 *g* for 10 min. The collected supernatant was stored at −80 °C. Samples of liver extracts containing the same amount of proteins were separated in SDS-PAGE on 7.5% acrylamide gels and transferred to PVDF membrane. Unspecific sites were blocked for 2 h with 5% Bovine Serum Albumin (BSA) in TBS Tween (20 mMTris/HCl, 500 mM NaCl, pH 7.5, 0.1% Tween 20) at 4 °C. The membranes were incubated with primary antibodies overnight at 4 °C, under gentle agitation. Primary antibodies against mouse monoclonal alpha tubulin (DM1A), mouse monoclonal anti-RECK, mouse monoclonal anti-eNOS, mouse monoclonal anti-Phospho-JNK (Thr183/Tyr185), mouse monoclonal anti-Phospho-p38 (Thr180/Tyr182), rabbit monoclonal anti-Phospho-ERK1/2 (Thr202/Tyr204), rabbit monoclonal anti-ERK1/2, rabbit polyclonal antibody anti-iNOS, rabbit polyclonal anti-JNK, and rabbit polyclonal anti-p38 were used at 1:1000 dilution. Rabbit polyclonal anti TIMP-1 and TIMP-2 were used at 1:200. Membranes were washed in TBS Tween (Na2HPO4 8 mM, NaH2PO4-H2O 2 mM, NaCl 140 mM, pH 7.4, 0.1% Tween 20) and incubated with peroxidase-conjugated secondary anti-Rabbit or anti-Mouse antibodies at a 1:2000 dilution. The membranes were then stripped and incubated with tubulin monoclonal antibody (1:5000) and subsequently with anti-mouse (1:10,000) to assess uniformity of gel loading. Anti-iNOS was purchased from Cayman Chemical (Ann Arbor, Michigan, USA). RECK and eNOS were bought from Santa Cruz Biotechnology. Mouse monoclonal antibody against TIMP-1 and TIMP-2 were purchased from Thermo Fisher Scientific (USA For the detection of MAPKs, the antibodies were obtained from Cell Signaling Technology [LS1] (Leiden, the Netherlands). Immunostaining was revealed with BIO-RAD Chemidoc XRS+ visualized using the ECL Clarity BIO-RAD (Milan, Italy). Bands intensity quantification was performed by BIO-RAD Image Lab Software™ 6.0.1., and autoradiograms showing statistically significant differences in terms of gel-loading homogeneity were excluded from the following biomarkers analyses.

### 2.6. Liver Histology

Liver biopsies were rapidly removed, fixed in 2% p-formaldehyde in 0.1 M phosphate buffer at pH 7.4 for 24 h and processed routinely until they were embedded in Paraplast wax. Sections were cut at 7 μm and stained with Hematoxylin and Eosin (H&E) for histological examination [16]

### 2.7. Statistical Analysis

Results are expressed as means value ± standard error (SE) for all data. The value of *p* < 0.05 was considered the criterion for statistical significance. To assess normality of variance changes, the Kolmogorov–Shapiro normality test was used. Data were analyzed by ANOVA with Tukey’s multiple comparison test as post-hoc test or Kruskall–Wallis and Dunn’s test, as appropriate. Statistical Analysis was performed using MedCalc Statistical Software version 18.11.3 (MedCalc Software bvba, Ostend, Belgium; https://www.medcalc.org; 2019).

## 3. Results

### 3.1. Transient Expression of Reck, Mmps, and Timps in a Rat Model of Hepatic I/R Injury

To study the expression of RECK under hepatic I/R, we used a rat model of partial I/R injury. After 60 min reperfusion, a time-dependent increase in serum levels of AST, ALT, ALP, and total and direct bilirubin was observed (Table 1). 

**Figure 1:**
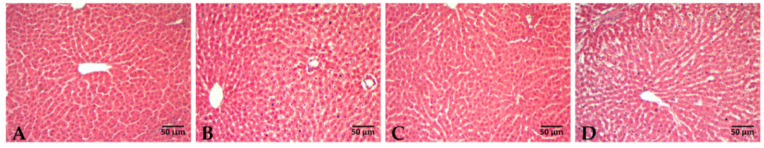
Liver histology at the end of reperfusion. Paraplast-embedded sections were cut at 7 μm and stained with H&E. Panel (**A**) and (**C**): 60/60 and 60/120 min sham-operated rats, respectively. Panel (**B**) and (**D**): rats submitted to ischemia followed by 60 or 120 min reperfusion, respectively.

This trend was supported by histological analysis (Figure 1). Livers from sham-operated animals showed well-preserved hepatic architecture while I/R caused marked injury to the parenchyma with sinusoid dilatation, extensive areas of cytoplasmic vacuolation, and wide areas of necrotic cells detached from the parenchyma, especially after 120 min reperfusion.

**Figure 2:**
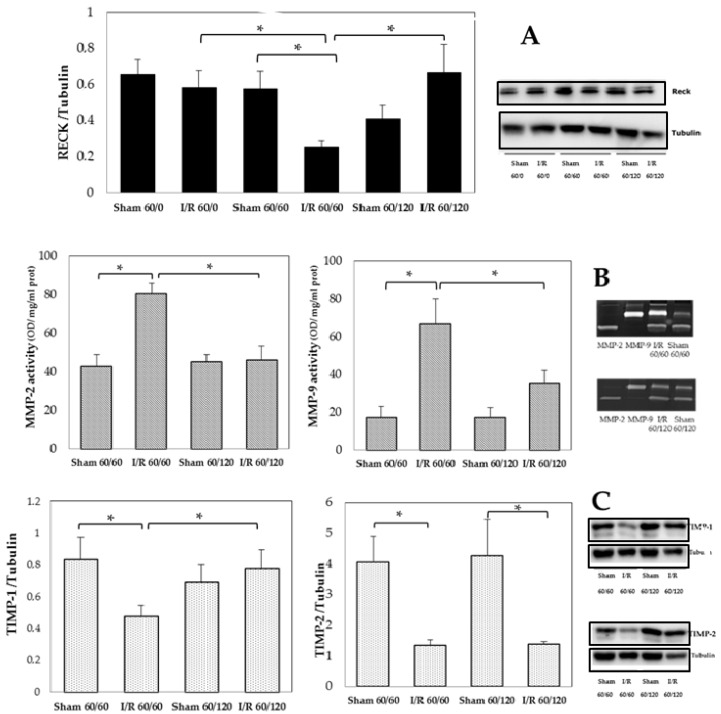
Hepatic MMP-2 and MMP-9 activity, RECK, TIMP-1, and TIMP-2 at the end of ischemia and after 60 min or 120 min of reperfusion. Panel (**A**): RECK, * *p* = 0.02; Panel (**B**): MMP-2, * *p* = 0.006; MMP-9: * *p* = 0.007; Panel (**C**): TIMP-1 * *p* = 0.04; TIMP-2, * *p* = 0.03. The results are reported as the mean ± SE of 6 different experiments. RECK, reversion-inducing cysteine-rich protein with Kazal motifs; TIMPs, tissue inhibitor of metalloproteinases.

The results of RECK protein analysis showed a significant decrease in RECK only at 60 min reperfusion when compared with 60 min ischemia or 120 min reperfusion or sham-operated groups; a complete recovery of RECK levels was found at 120 min reperfusion (Figure 2, Panel A). In addition, no changes in RECK content at the end of ischemia occurred when compared with sham-operated group. The opposite trend occurred for both MMP-2 and MMP-9 activities that increased after 60 min reperfusion when compared with sham-operated rats followed by a significant decrease in both hepatic MMP activities detectable at 120 min reperfusion comparable with those found in sham-operated rats (Figure 2, Panel B). The analysis of TIMP-1 showed a decrease after 60 min reperfusion but was re-expressed at 120 min reperfusion relative to the 60 min time point. No differences in expression between TIMP-2 were detected between 60 min versus 120 min reperfusion (Figure 2, Panel C).

### 3.2. Transient Expression of Mapks under Hepatic I/R 

**Figure 3:**
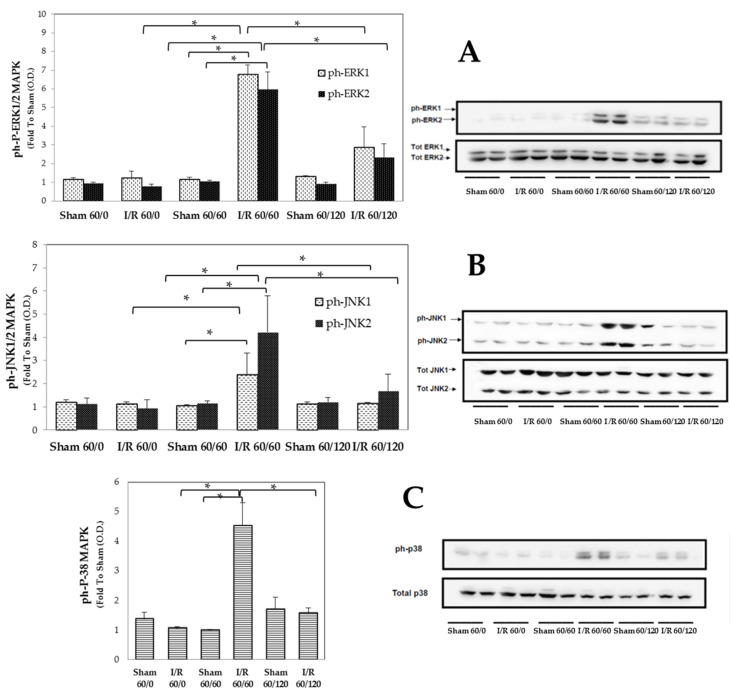
Hepatic MAPK pathways (ERK1/2, JNK1/2 and p38) at the end of ischemia and after 60 min or 120 min reperfusion. Panel (**A**): ERK1, * *p* = 0.025; ERK2, * *p* = 0.025; Panel (**B**): JNK1, * *p* = 0.028; JNK2, * *p* = 0.043; Panel (**C**): P-38, * *p* = 0.014. The results are reported as the mean ± SE of 6 different experiments.

Animals that had undergone 60 min ischemia followed by 60 min reperfusion displayed higher phosphorylation of MAPKs (ERK1/2, JNK1/2 and p38) expression than ischemia or sham-operated animals (Figure 3 A–C), suggesting an early enhancement of the activation of these pathways by I/R injury. On the contrary, the longer I/R challenge resulted in lower expression levels of the MAPK phosphorylated forms, with values similar to those recorded in sham-operated animals, thus indicating a time-dependent quenching of I/R-injury. No significant changes in expression of total MAPK isoforms were recorded in ischemia group as well in sham-operated rats.

### 3.3. Changes in Enos and Inos Expression Under Hepatic I/R

**Figure 4:**
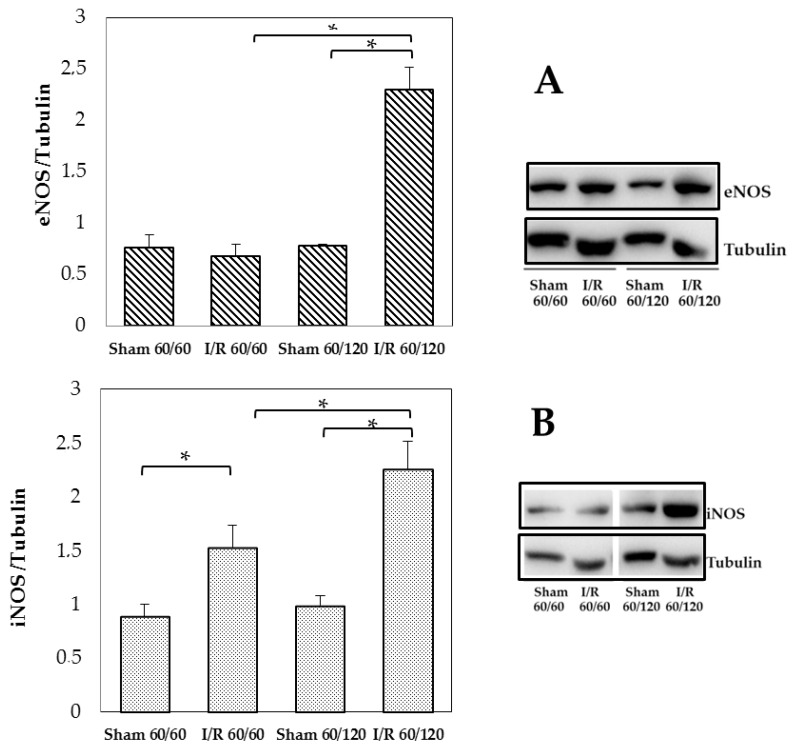
Hepatic eNOS and iNOS at the end of reperfusion. Panel (**A**): eNOS, * *p* = 0.002; Panel (**B**): iNOS, * *p* = 0.004. The results are reported as the mean ± SE of 6 different experiments.

At 120 min reperfusion, a significant increase in eNOS expression was found and it was about 3-fold compared with sham-operated or 60 min reperfused animals (Figure 4, Panel A). Changes in iNOS expression emerged comparing sham and I/R treated groups both after 60 min or 120 min reperfusion: a time-dependent increase in iNOS content was found (Figure 4, Panel B).

## 4. Discussion

The present data provide new insights into the underlying molecular mechanisms that occur during reperfusion in hepatic I/R injury: a transitory decrease in RECK and TIMPs, concomitant with an increases in both MAPK and MMP activity, suggests their role as triggering factors of the organ dysfunction.

As previously documented, the kinetics of the reperfusion injury may also affect the entity of the expression and activation of MAPK signaling pathways involved in the I/R-induced damage [17]. A crucial intracellular step that can counteract the deleterious effects of I/R injury is the selective activation of MAPK. A time-course experiment of MAPK expression after liver I/R showed an increase in phosphorylation of each MAPK (ERK1/2, JNK1/2, and p38), detectable after 60 min reperfusion and the activation decreased after 3 h reperfusion, especially for JNK [18]. Here we confirmed the early and transient MAPK activation, coming back to values similar to those recorded in basal condition after 120 min reperfusion. 

In keeping with the data on MAPK activation, when we measured MMP-2 and MMP-9 activity as well as the expression of their respective TIMP physiological inhibitors in the liver homogenates, we found a parallel between the early and transient activation of MMP-2 and MMP-9 and that observed for MAPKs, suggestive of simultaneous contribution to I/R-induced protein expression changes. 

Here, for the first time, we demonstrated that both MMP-2 and MMP-9 over activation at 60 min was associated with RECK down-regulation, whereas the up-regulation of RECK expression was recorded at 120 min when no significant MMP activation was detectable. It has been reported that RECK overexpression decreases the amount of active MMP-2 and MMP-9 in conditioned medium and inhibits metastatic activity in vitro [19] and in vivo [12]. The evaluation of genotypes among hepatocellular carcinoma (HCC) patients suggested a possible involvement of RECK gene rs11788747 polymorphism in increasing the susceptibility of individuals to HCC [20]. In addition, RECK expression was found to be reduced in kidney epithelial cells subjected to hypoxia [21]. Here, for the first time, we demonstrated that modulation of RECK expression following liver I/R injury was paralleled by changes in MMP-2 and MMP-9 activation, thus suggesting a potential cross-talk mechanism linking these two signaling cascades. Our results were supported by findings reported in a recent study using a rat model of cerebral ischemia [22]. Besides, we did not measure activation of signaling cascades (neither at the very beginning of reperfusion, nor in non-ischemic liver lobes; thus, we cannot offer a comprehensive understanding here of all the kinetics factors potentially affecting their expression and activation. MMPs and TIMPs play also an important function in the preservation of liver homeostasis [23]. In particular, the major endogenous regulator of MMP-9, TIMP-1, exerts a protective role in the control of the survival of liver cells during I/R injury [24]. Curzio et al. reported that TIMP-1 was induced following liver I/R with an earlier induction so we can hypothesize that the failure to recover TIMP-2 at 120 min is due to slower induction times [25]. In addition, TIMP-1 has been reported to play the major protective role: the inability of TIMP-1^−/−^ mice to express TIMP-1 resulted in enhanced liver damage and in lethal hepatic I/R injury [24]. Our data support these results: an opposite trend was found comparing MMP-9 activation versus TIMP-1 content.

RECK downregulation under hypoxic conditions has been demonstrated to be mediated by involvement of ERK1/2, JNK, and p38 MAPK signaling pathways [26]. The same authors demonstrated that inhibition of the MAPK signaling pathways restores RECK expression and its ability to affect MMP activity. In particular, activation of the p38 pathway and p38-associated inflammatory processes play a crucial role in post-ischemic damage [27]. Although several studies have demonstrated a detrimental role of JNK activation in I/R injury [28], the results about a protection by JNK inhibition are still controversial [27,29]. There are also studies indicating the potential involvement of ERK 1/2 in hepatic I/R injury [30]; to date, the potential role of ERK1/2 as a therapeutic target for liver I/R injury has not been clarified yet, because the molecules used are also able to modulate other pathways different from ERK1/2 [28]. However, liver protection against I/R injury was recently obtained via suppressing the MAPK signaling [1]. Although we demonstrated a time-dependent effect of reperfusion injury on the cross-talk mechanisms linking RECK expression to MAPK activity, we cannot rule out the potential impact of the kinetics of the ischemia injury on the activation/expression of the tested signaling cascades. In fact, as shown by Cursio and colleagues [18], a longer kinetics of ischemia (120 min of normothermic liver ischemia) resulted in sustained p38 MAPK activation until 3 h reperfusion, whereas, in keeping with our findings, ERK1/2 degree of activation already decreased after 1 h reperfusion. 

We also showed no significant differences in RECK and MAPK content between ischemic livers without reperfusion and either the sham-operated and 120 min reperfusion groups, thus confirming that activation of the signaling cascades is mainly related to the reperfusion injury in our experimental conditions.

Once the MAPK pathway is activated, MAPKs phosphorylate downstream protein kinases and transcription factors, resulting in upregulation of both iNOS and eNOS. Under normal conditions, only eNOS is present in the liver, and low levels of NO regulate the hepatic perfusion [31]. Alternatively, the excess production of NO, generated primarily by iNOS, has been implicated as a mediator of cellular injury at sites of inflammation, including liver I/R injury [32]. Under these circumstances, NO reacts with molecular oxygen or superoxide and generates reactive nitrogen species, which may interfere with many biological functional and structural processes of liver injury evoked by hepatic I/R, including ECM degradation [10]. Here, we confirmed that the early MAPK activation resulted following overexpression of iNOS and eNOS, leading to excessive NO production, which contributes to the liver damage [33] and also remote organs [34]. Besides, specific iNOS inhibition has been demonstrated to down-regulate MMP-9 activity and disrupts leukocyte migration in hepatic I/R injury [10]. These findings further support the mechanistic loop linking MAPKs to MMPs here described. However, only the use of drugs that specifically inhibit these signaling cascades may allow to identify a causal relationship between these two pathways and their modulation by RECK. Unfortunately, the lack of knowledge of the in vivo pharmacological profile of highly selective pharmacological inhibitors limits further investigation.

## 5. Conclusions

In conclusion, we demonstrated that the kinetics of reperfusion selectively affect the activation of signaling pathways involved in hepatic I/R injury. Both MAPKs and MMPs showed an early and transient activation, which contribute to the development of organ dysfunction and injury. Our data show that reperfusion-induced modulation of RECK expression is paralleled by opposite changes in MAPK activation and MMP activity. These findings may contribute to better understanding intracellular pathways cross-regulation sensitive to liver I/R injury and, thus, to providing the rationale for much needed novel agents to fill the current therapeutic gaps. However, further studies are needed to better clarify the reciprocal interaction of these pathways for a wider understanding of the intricate network of cellular and functional interactions leading to I/R-related liver injury.

## Figures and Tables

**Table 1:** Serum levels of AST, ALT, ALP (U/L), and total and direct bilirubin (mg/dL).

	Sham 60/60	I/R 60/60	Sham 60/120	I/R 60/120
AST	243 ± 57	3444 ± 1062	198 ± 43	10,387 ± 1158 *
ALT	66 ± 19	3830 ± 961	61 ± 28	9320 ± 1040 *
ALP	431 ± 50	605 ± 51	417 ± 55	769 ± 29 *
Total Bilirubin	0.13 ± 0.024	0.25 ± 0.070	0.12 ± 0.011	0.35 ± 0.043
Direct Bilirubin	0.045 ± 0.019	0.17 ± 0.032	0.04 ± 0.024	0.18 ± 0.013

Aspartate transaminase, AST; alanine transaminase, ALT; alkaline phosphatase, ALP; * *p* < 0.05.
